# Gene expression in the chicken caecum is dependent on microbiota composition

**DOI:** 10.1186/s13567-017-0493-7

**Published:** 2017-12-04

**Authors:** Jiri Volf, Ondrej Polansky, Zuzana Sekelova, Philippe Velge, Catherine Schouler, Bernd Kaspers, Ivan Rychlik

**Affiliations:** 1 Veterinary Research Institute, Hudcova 70, 621 00 Brno, Czech Republic; 2 ISP, INRA, Université François Rabelais de Tours, 37380 Nouzilly, France; 3 Department for Veterinary Sciences, Institute for Animal Physiology, Faculty of Veterinary Medicine, Ludwig-Maximilians-Universität München, Veterinastr. 13, 80539 Munich, Germany

## Abstract

Gut microbiota is of considerable importance for each host. Despite this, germ-free animals can be obtained and raised to sexual maturity and consequences of the presence or absence of gut microbiota on gene expression of the host remain uncharacterised. In this study, we performed an unbiased study of protein expression in the caecum of germ-free and colonised chickens. The major difference between these two groups was in the expression of immunoglobulins which were essentially absent in the germ-free chickens. Microbiota also caused a minor decrease in the expression of focal adhesion and extracellular matrix proteins and an increase in the expression of argininosuccinate synthase ASS1, redox potential sensing, fermentative metabolic processes and detoxification systems represented by sulfotransferases SULT1C3 or SULT1E1. Since we also analysed expression in the caecum of *E. coli* Nissle and *E. faecium* DSM7134 mono-associated chickens, we concluded that at least immunoglobulin expression and expression of cystathionine synthase (CBS) was dependent on microbiota composition with *E. coli* Nissle stimulating more immunoglobulin and PIGR expression and *E*. *faecium* DSM7134 stimulating more CBS expression. Gut microbiota and its composition therefore affected protein expression in the chicken caecum though except for immunoglobulin production, the remaining differences were unexpectedly low.

**Electronic supplementary material:**

The online version of this article (10.1186/s13567-017-0493-7) contains supplementary material, which is available to authorized users.

## Introduction

Complex gut microbiota is of considerable importance for its host. Microbiota affects food or feed digestion [1], restricts pathogen growth or pathogen adhesion to the intestinal cell surface [2], or produces metabolites which cannot be synthesized by its host. It is therefore not surprising that many reports on the association of specific microbiota with particular disorders have been published [3, 4]. Despite this, germ-free mice, rats, pigs or chickens can be raised, which means that at least in these species the host is not strictly dependent on the presence of microbiota in the intestinal tract. Mice can be kept as germ-free even throughout their whole life including reproduction which partly contradicts the frequency of reports associating microbiota with different disorders. Basal gut functions must be therefore secured even in the absence of microbiota and the differences in gene expression of the host may not be as extensive as one would expect. On the other hand, this does not mean that germ-free animals remain unaffected. As was reviewed by Furuse and Ocamura, germ-free chickens showed faster growth, thinner intestinal wall or higher urea/uric acid ratio in faeces [5]. Many germ-free models are characterised by a megacaecum or megacolon [6] and metabolism and recycling of bile acids is also affected in the absence of gut microbiota [7, 8].

Animals, including chickens, do respond to gut colonisation. The chicken intestinal tract responds to colonisation by transient induction of an inflammatory response [9] which may signal to the infiltrating lymphocytes [10, 11]. Moreover, we recently described multiple genes with increased or decreased expression in the caecum of newly hatched chickens following oral inoculation with microbiota from adult hens [12]. However, especially in the latter study it was rather difficult to differentiate to what extent the chicken response was to microbiota colonisation itself and/or to sudden change in the conditions in the caecum after oral administration of 10^9^ different bacteria. In this study, we therefore determined gene expression in germ-free, conventional, and *E. coli* and *Enterococcus faecium* mono-associated chickens up to the age of 56 days of life. Testing gene expression in nearly 2-month-old chickens permitted identification of gene expression which was dependent on the presence or absence of microbiota and not on a sudden change from sterile to heavily colonised intestinal tract. Mono association of chickens with *E. coli* and *E. faecium* also meant that differences in protein/gene expression of chickens colonised exclusively by a representative of Gram-negative or Gram-positive microbiota could be addressed. The results we obtained were somewhat unexpected. Only immunoglobulin expression was strictly dependent on the presence of live bacteria in the caecum. Besides immunoglobulins, we found differences also in the expression of proteins involved in sensing redox potential, detoxification, arginine metabolism and assembly of the extracellular matrix but the range of induction or suppression of these genes in response to the presence of gut microbiota was two logs lower than that of immunoglobulins.

## Materials and methods

### Experimental chickens

Leghorn White chickens originated from the Specific-Pathogen-Free (SPF) flock reared at the infectiology platform PFIE (INRA Val de Loire). The germ-free chickens were obtained by hatching and rearing chickens under sterile conditions as follows. The eggs were collected immediately after laying and surface was sterilized by immersion in 1.5% Divosan Plus VT53 (Johnson Diversey, France) for 5 min before transferring into a sterile HEPA-filtered incubator. After 18 days, the surface of the eggs was sterilized in 1.25% Divosan for 4 min at 37 °C again. After hatching, the temperature was maintained at 37.5 °C for the first week of life and then reduced by one degree per day to a stable temperature of 25 °C. Chickens were offered ad libitum X ray-irradiated starter diet from Special Diets Services (Dietex, Argenteuil, France) and sterilized water for the entire duration of the experiment (56 days). The sterility of chickens was confirmed weekly by taking fresh fecal droppings which were incubated in tubes containing 10 mL of sterile brain–heart infusion broth under both aerobic and anaerobic conditions. Culture in this medium allows the growth of bacteria, yeast and fungi. At the end of the experiment, the presence of non-culturable bacteria in fecal and caecal samples of germ-free chickens was tested by quantitative PCR using primers corresponding to “all bacteria” [13].

The experiments were performed on two independent occasions. In the first experiment, there were 3 groups of 5 chickens kept in isolators. The first group was kept non-inoculated, i.e. germ-free. The second group was inoculated with a mixture of *E. coli* Nissle1917, *Enterococcus faecium* DSM 7134 (*E. faecium*)*, Lactobacillus rhamnosus* DSM 7133 and *Clostridium butyricum* DSM 10702 (hereafter referred to as tetraflora). The third group was hatched and reared under conventional conditions. Experiment 1 was terminated when the chickens were 56 days old. In experiment 2, chickens in group 1 were orally inoculated with heat-killed tetraflora. Heat inactivated tetraflora was administered 3 times a week to each chicken until the end of the experiment. Chickens in groups 2 and 3 were mono-associated with one of two strains present in tetraflora, with Gram-negative *E. coli* Nissle1917 or Gram-positive *E. faecium* DSM 7134, respectively. Chickens in both experiments were inoculated with 10^7^ CFU of appropriate bacterium or mixture in 0.2 mL inoculum. The last group of chickens was represented by germ-free controls. There were 15 chickens housed in isolators in each group in the beginning of experiment 2. Five chickens from each group were sacrificed on days 14, 28 and 56 of life in experiment 2 (Table 1). In both experiments, samples of cecal tissue were collected into RNALater during post mortem analysis and stored at −20 °C until mRNA and protein purification. In experiment 2, the caecal contents of all chickens sacrificed on day 56 were collected also to determine chicken proteins secreted and adhered to *E. coli* or *E. faecium*.

**Table 1:** **List of samples processed in this study**

		Sample type	Germ-free	Conventional	Tetraflora	*E. coli*	Enterococcus	Killed
Exp1	Day 56	Caecal tissue	√	√	√			
Exp2	Day 14	Caecal tissue	√			√	√	√
Exp2	Day 28	Caecal tissue	√			√	√	√
Exp2	Day 56	Caecal tissue	√			√	√	√
Exp2	Day 56	Caecal content	√			√	√	

### Protein and RNA purification from chicken cecal tissue

Samples of chicken cecal tissue (50–100 mg) were used for parallel protein and RNA isolation. The samples were recovered from RNALater storage, mixed with 1 mL of TRI Reagent (MRC) and homogenized with a MagNA Lyser (Roche). Fifty microlitre of bromoanisole was added to the homogenate and after centrifugation at 14 000 *g* for 15 min, proteins captured in the lower phenolic phase were precipitated with acetone. RNA present in the upper aqueous phase was collected (500 μL) and mixed with an equal volume of 70% ethanol. This mixture was applied onto RNeasy purification columns. Subsequent washing and purification steps were performed with RNeasy Mini Kit exactly as recommended by the manufacturer (Qiagen). One μg of RNA was immediately reverse transcribed into cDNA using M-MLV reverse transcriptase (Invitrogen) and oligo (dT) primers.

Chicken proteins adsorbed at the surface of colonising *E. coli* and *E*. *faecium* were detected exactly as described previously [12]. In brief, the cecal contents (50–100 mg) were resuspended in 2 mL of 0.1% Tween 80, homogenized and centrifuged for 1 min at 50 *g* to remove coarse particles. Supernatant was transferred to a new tube and centrifuged at 4000 *g* for 10 min and bacterial pellet was washed 5 times with 0.1% Tween 80. After the last washing step, the pellet was resuspended in 100 μL of 1% SDS and incubated at 100 °C for 1 h. Subsequently, the protein lysate was mixed with 1.5 mL of TRI Reagent and processed for protein purification as described above.

### Protein mass spectrometry

Acetone precipitated protein pellets were dissolved in 300 μL of 8 M urea and processed following to the FASP protocol [14] using 10 kDa MWCO Vivacon 500 filtration device (Sartorius Stedim Biotech). Initial protein washing was performed with 8 M urea followed by centrifugation for 12 min at 12 000 *g*. The reduction of disulfide bonds was performed with 10 mM dithiothreitol for 15 min at room temperature and acetylation was done with 50 mM iodoacetamide for 15 min at room temperature. After 3 washes with 10 mM triethylammonium bicarbonate, trypsin (Promega) was added at a 1:50 ratio and the digestion proceeded for 16 h at 30 °C.

Proteins were quantified by 2 different protocols, either using stable isotope dimethyl labelling protocol or by label free analysis. When using dimethyl labelling protocol, samples were pooled and a single ratio of protein abundance in experimental and control group was obtained. On the other hand, label free analysis was performed for each sample individually thus allowing for statistical analysis.

In stable isotope dimethyl labelling protocol analysis, total peptide concentration was determined spectrophotometrically (Nanodrop, Thermo Scientific) in each sample and samples from all germ-free and all conventional chickens in the experiment 1 were pooled. Pooled samples were then labelled using the stable isotope dimethyl labelling protocol [14], mixed and 3 subfractions were prepared using Oasis MCX Extraction Cartridges (Waters). The subfractions were desalted on SPE C18 Extraction Cartridges (Empore) and concentrated in a SpeedVac (Thermo Scientific) prior to LC–MS/MS. This analysis was performed only for pooled samples from all germ-free and all conventional chickens collected in the experiment 1.

LC–MS/MS analysis of both labelled and label-free tryptic peptides was performed using a Dionex UltiMate 3000 RSLC liquid chromatograph connected to a LTQ-Orbitrap Velos Pro hybrid mass spectrometer (Thermo Scientific). For each analysis, 5 μg of peptide sample was used. Each sample was separated on an EASY Spray C18 column (length 25 cm, I.D. 75 μm, particles 3 μm) using 300 nL/min flow rate of solvent A (0.1% formic acid) and solvent B [0.1% formic acid in 20/80 H_2_O/ACN (vol/vol)] and 150 min long reverse-phase gradient with concentration of solvent B gradually increasing from 4 to 40%. From MS spectra (Orbitrap analyzer, 30 000 FWHM resolution, mass range 390–1700 *m/z*), the 10 most intensive peptides were fragmented using CID fragmentation (normalized collision energy 35) followed by MS/MS scan (LTQ analyzer). Raw LC–MS/MS data were analyzed using Proteome Discoverer v1.4. MS/MS spectra identification was performed by the SEQUEST algorithm using chicken protein sequence database. Precursor and fragment mass tolerance were 10 ppm and 0.5 Da respectively. Carbamidomethylation (C) and oxidation (M) were set as static and dynamic modifications, respectively. Dimethylation (N-term and K) was set as static modification in the label-based analysis. Only peptides with a false discovery rate ≤ 5% were considered.

### Protein quantification

To identify differently expressed proteins, the following workflow was adopted. Using data from label-free analysis, peptide spectrum matches (PSMs) counts were used to calculate protein abundance as a percentage in each sample. These values were used to identify proteins with significantly different abundance in the caeca of experimental and germ-free chickens by t test. The percentage abundance was used also for the calculation of fold increase or decrease of individual proteins. In a parallel procedure with data from label-free analysis, proteins in each sample were ranked in descending order according to peak area and peak areas were replaced with ranking. These numbers were then used to identify differently ranking proteins in the caeca of experimental and germ-free chickens by *t* test. Only the proteins with significant differences in both PSM percentage abundance and peak area ranking were considered as differently abundant in response to colonisation. This set of proteins was finally compared with proteins quantified by stable isotope dimethyl labelling and only the significantly abundant proteins in label-free analysis with the same direction in change of abundance in stable isotope dimethyl labelling analysis were finally considered as differently expressed. The set of differentially abundant proteins was tested using STRING database (v10) to reveal significantly overrepresented KEGG categories [15].

### Quantitative reverse transcribed PCR (qRT-PCR)

cDNA was diluted 10 × with sterile water prior to real-time PCR. qRT-PCR was performed in 3 μL volumes in 384-well microplates using QuantiTect SYBR Green PCR Master Mix (Qiagen) and a Nanodrop pipetting station (Inovadyne) for PCR mix dispensing. PCR and signal detection were performed using a LightCycler 470 (Roche) with an initial denaturation at 95 °C for 15 min followed by 45 cycles of 95 °C for 20 s, 60 °C for 30 s and 72 °C for 30 s. Each sample was subjected to real-time PCR in duplicate and the mean values of the duplicates were used for subsequent analysis. The Ct values of the genes of interest were normalized (ΔCt) to a geomean Ct value of 3 reference genes, TBP1, HMBS and ADA and the relative expression of each gene of interest was calculated as 2^−ΔCt^. The house-keeping reference genes were chosen out of 9 candidates using NormFinder software [16]. All the primers are listed in Additional file 1.

qRT-PCR data were analysed by comparing expression profiles of differently colonised chickens to the expression in germ-free chickens of the same age and from the same experiment. However, since the qRT PCR was quite robust, we also compared data from all germ-free chickens (i.e. irrespective of age or experiment) to all chickens inoculated with particular microbiota (i.e. irrespective of their age). Due to the higher “n” in the summed data (20 germ free chickens compared to 15 chickens colonised with *E. coli* or *E*. *faecium* or heat killed tetraflora, or 5 chickens colonised tetraflora or 5 conventional chickens), we gave a higher weight to the latter type of analysis, though this type of testing was always compared with age and experiment dependent comparisons not to overlook age-dependent expression patterns. In all cases, *t* test comparison to the values obtained from germ-free chickens was used for statistical analysis.

### Ethical statement

Animal experiments performed with germ-free, inoculated and conventional chickens were carried out in strict accordance with French legislation. The experiments have been approved by the “Ministère de l’éducation nationale, de l’enseignement superieur et de la recherche” under the Protocol No. APAFIS#5833-20l60624l6362298 v3. The principles of Reduction, Replacement and Refinement were implemented in all these experiments.

## Results

### Identification of differently expressed proteins in response to colonization by protein mass spectrometry

In experiment 1 (conventional and tetraflora colonised chickens), we identified 6302 different proteins expressed in the caecum of 56-day-old chickens. In the second experiment (*E. coli* and *E. faecium* associated chickens), we identified 6941 proteins in the caecum of 14-day-old chickens, 7685 proteins in 28-day-old chickens and 7831 proteins in 56-day-old chickens. Due to minor variation among the two experiments, protein expression had to be compared between inoculated chickens and appropriate germ-free controls of the same age and from the same experiment. After adopting all selection criteria, 149 proteins were differentially expressed in at least one experimental group inoculated with live bacteria compared to expression in germ-free chickens (Table 2 and Additional file 2).

**Table 2:** **List of 25 of the most induced or suppressed proteins in the chicken caecum following colonisation with microbiota of different composition in comparison to protein abundance in the caecum of germ-free chickens** (only the significant fold changes to the expression in germ free chickens are shown)

Microbiota INDUCED caecal proteins										Microbiota SUPPRESSED caecal proteins									
Protocol	Label-free								Label		Label-free								Label
Microbiota	*E. coli*			*Enterococcus*			Tetra	Conv	Conv		*E. coli*			*Enterococcus*			Tetra	Conv	Conv
Age (days)	14	28	56	14	28	56	56	56	56		14	28	56	14	28	56	56	56	56
Ig λ		6.61	8.77		2.67		7.30	8.00	20.00	PCNA	1.79								20.00
GRPEL1						1.41			20.00	GSTK1							2.44		3.93
PRSS2					1.72			2.92	17.59	IVD					1.82				3.62
CLIC5				1.76		1.81			15.53	COL6A3		2.72	3.39			6.21			2.50
RPL14					1.44				9.59	UCHL1					1.38				2.44
TXN							1.79		8.40	COL6A2						4.81			2.34
HDGF						1.52			7.83	BAG2					1.61				2.29
CATHL2		2.50							4.12	BASP1								3.99	2.25
PLS1								2.63	3.49	COL6A1		2.02	1.91			4.28			2.20
PACSIN1		1.61							3.21	MYH10			2.07			3.29			2.19
SULT1C3	1.23							1.85	2.95	ACTA1			1.49						2.06
SYNJ2BP		2.04			1.73				2.57	SYNM		1.71							2.03
PAPSS2	1.70								2.49	PYGB		1.74	2.21			4.34			2.01
B2 M			1.58						2.47	PLEKHC1			2.21			5.95			1.95
ASS1								3.57	2.28	POSTN		1.59			1.79				1.78
CALB1								3.04	2.26	SARNP				1.35				1.65	1.75
PSAP	1.95					3.17			2.18	YWHAQ		1.30							1.74
TXNL1							1.44		2.08	EIF4A2		1.61	3.55			6.45			1.71
ISG12-2		1.42							2.00	VAT1					1.41				1.70
PON2					2.64				1.85	MYH11			1.79			2.71			1.67
CDH17		1.19				1.79			1.81	TPPP3			1.24			1.22			1.67
MPP1		1.61	2.26	2.23	2.00	2.66	2.08		1.81	TLN1						3.37			1.66
HSD17B4							2.02		1.78	DPYSL2			1.24						1.61
TST		1.33			1.40	1.20			1.77	COL5A1							2.49	3.69	1.61
CNN3			1.44			1.55			1.75	ATP1A1						3.33			1.60

### Immunoglobulin expression is dependent on the presence of live microbiota

**Figure 1:**
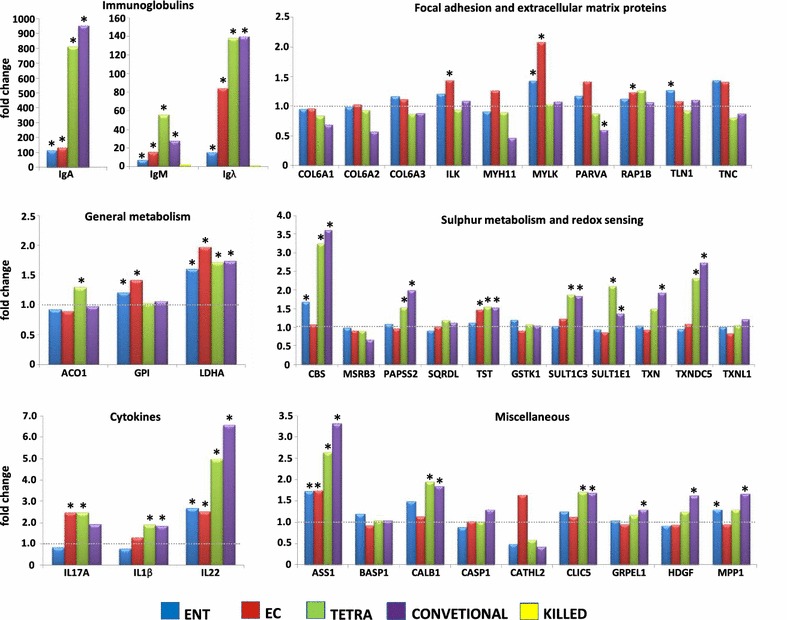
**Differential expression of 39 selected genes in the chicken caeca following their colonisation with microbiota of different composition.** Each column indicates fold induction (or suppression) in comparison to the expression of particular gene in germ-free chickens determined by qRT PCR. ENT, chickens colonised with *E. faecium*, EC, chickens colonised with *E. coli*; TETRA, chickens colonised with tetra flora; CONVENTIONAL, chickens colonised with conventional microbiota; KILLED, chickens treated with killed tetraflora (tested only for immunoglobulin transcription). Fold changes and statistical significances shown in this figure were calculated from average expressions in all germ free, *E. coli*, *E. faecium*, tetraflora or heat killed tetraflora inoculated chickens, as well as all conventional chicken irrespective of their age. *Significantly induced or suppressed genes in comparison to their expression in germ-free chickens (t test, *p* < 0.05).

Out of the differentially expressed proteins, expression of immunoglobulins exhibited the greatest difference in production in germ-free and colonised chickens. Mass spectrometry showed that immunoglobulins were induced by conventional microbiota, tetraflora, *E. coli* Nissle or *E. faecium* DSM7134 but not by heat killed tetraflora. To verify data obtained by protein mass spectrometry, IgA, IgM and Igλ expression was tested also at the transcriptional level by qRT PCR. Similar to protein mass spectrometry, transcription of immunoglobulin genes was dependent on live gut microbiota since significantly higher levels of immunoglobulin transcripts were recorded in the colonised chickens compared to germ-free chickens or chickens administered heat-killed tetraflora (Figure 1). Out of live bacteria, tetraflora and conventional microbiota stimulated transcription of IgA, IgM as well as Igλ light chain more than *E. coli* Nissle, and *E. coli* stimulated immunoglobulin transcription more than *E. faecium* DSM 7134. There was therefore a clear microbiota composition dependent effect and Gram-positive *E. faecium* DSM 7134 was the least stimulatory bacterial species of all compared.

### Functional classification of additional proteins induced or suppressed by gut microbiota

Since the inoculation of chickens with killed microbiota did not induce immunoglobulin expression (Figure 1), we considered this intervention as having minimal effect on protein expression in the chicken caecum and we therefore excluded this group from further considerations. Classification of the remaining proteins which were induced or repressed by live bacteria into functional categories using STRING showed that differentially abundant proteins belonged to pathways such as focal adhesion, microbial metabolism in diverse environments, sulphur metabolism, ribosome, ECM-receptor interaction, glycolysis/gluconeogenesis, biosynthesis of amino acids, metabolic pathways, starch and sucrose metabolism, and cysteine and methionine metabolism. When we separately tested only the proteins of increased abundance in the colonised chickens, these were classified into the categories sulphur metabolism, microbial metabolism in diverse environments, cysteine and methionine metabolism, metabolic pathways and spliceosome. On the other hand, proteins from categories focal adhesion, ECM-receptor interaction and cell cycle were present in lower abundance in the colonised chickens compared to germ-free chickens.

### qRT PCR verification of gene expression at the level of transcription

Next, we used qRT PCR to confirm protein mass spectrometry data also at the RNA level. qRT PCR verification was performed for selected genes involved in sulphur metabolism, glycolysis and focal adhesion, and in a group of genes encoding proteins that were either induced in multiple groups of differently inoculated chickens or which exhibited unexpectedly high differential abundance at protein level in at least one of the tested groups. Finally, we tested the expression of IL1β, IL17 and IL22 cytokines which are known to respond to gut colonisation [9].

Out of the “glycolytic” genes (ACO1, GPI and LDHA), LDHA gene expression was induced by conventional microbiota, tetraflora, *E. coli* and *E. faecium*. Expression of GPI was induced by *E. coli* and *E. faecium*, and ACO1 gene expression was induced by tetraflora. Within the “focal adhesion” genes (COL6A1, COL6A2, COL6A3, ILK, MYH11, MYLK, PARVA, RAP1B, TLN1 and TNC), though there were several statistically significant differences in their expression in the colonised and germ-free chickens, the pattern and range of these differences did not follow any clear logic (Figure 1). We therefore concluded that the expression of these genes was not dominantly regulated at the RNA level.

PAPSS2, SQRDL, CBS, TST and MSRB3 represent genes directly associated with sulphur metabolism. In addition, we quantified also transcription of SULT1C3, SULT1E1, GSTK1, TXN, TXNDC5 and TXNL1 genes which are involved in sulphur-dependent detoxification or redox potential sensing. CBS, PAPSS2, TST, SULT1C3, SULT1E1 and TXNDC5 were induced by conventional microbiota and tetraflora. In addition, CBS gene expression was induced also by *E. faecium* and TST was induced also in *E. coli*-associated chickens (Figure 1).

Out of the proteins which were induced in multiple groups of differently inoculated chickens or which exhibited high differential abundance at the protein level (CALB1, MPP1, ASS1, GRPEL1, CLIC5, HDGF, CATHL2, CASP1 and BASP1), conventional microbiota and tetraflora induced ASS1, CALB1 and CLIC5 also at the RNA level. ASS1 was induced also by *E. coli* or *E. faecium*. GRPEL1, HDGF and MPP1 were significantly induced by conventional microbiota and MPP1 was induced also by *E. faecium* (Figure 1).

The last set of qRT PCR was targeted at the expression of IL1β, IL17 and IL22 cytokines. IL22 expression was induced by live bacteria of any composition out of which *E. faecium* was the least stimulatory. IL1β expression was induced by conventional microbiota and tetraflora, and IL17 expression was significantly induced by tetraflora and *E. coli*.

### Identification of proteins present in caecal contents of germ-free, *E. coli* and *E. faecium* associated chickens

**Figure 2:**
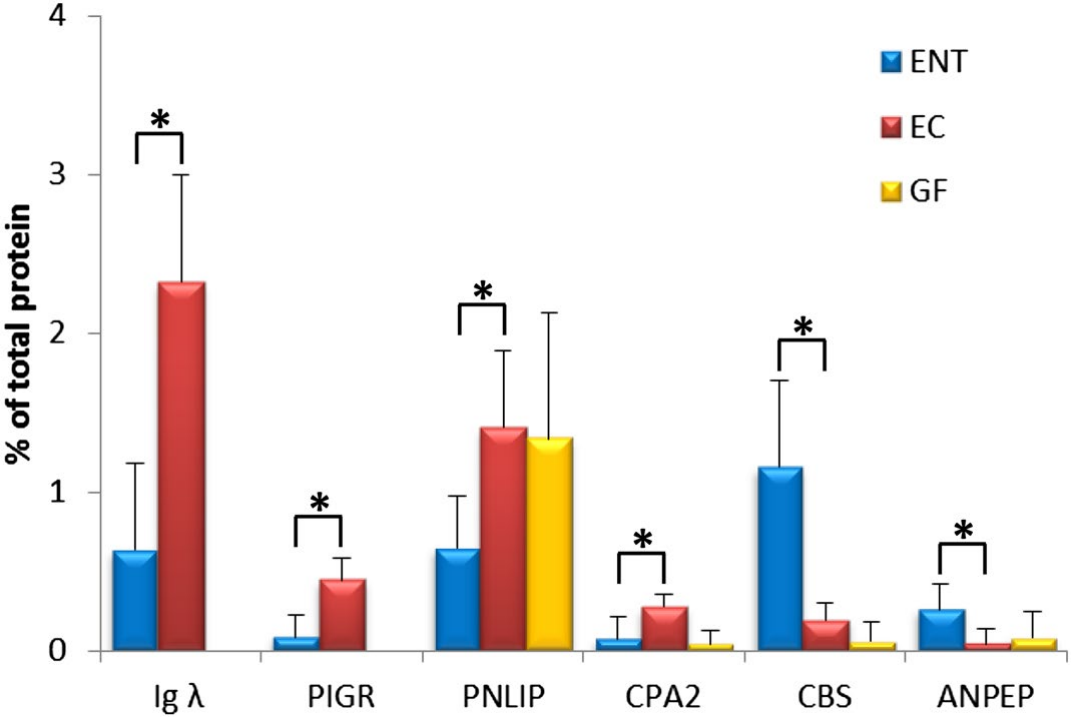
**Proteins adsorbed to the pellets obtained from caecal contents of germ-free,** ***E. coli*** **and** ***E. faecium*** **associated chickens.** Six proteins were found as differently abundant either in *E. coli* or *E. faecium* associated chickens (*p < 0.05). Igλ, PIGR, pancreatic lipase PINLIP and CPA2 carboxypeptidase A2 were more abundant in the pellets obtained from caecal contents of *E. coli* associated chickens. Cystathionine β-synthase CBS and ANPEP carboxypeptidase N were more abundant in the pellets obtained from the chickens associated with *E. faecium.*

Secreted immunoglobulins can be found as adsorbed to gut microbiota [12]. Finally, we tested whether differences in gene expression in the caecal tissue may result also in different interactions of chicken proteins with gut microbiota. This analysis was performed only for the caecal contents of germ-free, *E. coli* and *E. faecium* associated chickens on day 56 of experiment 2. In total, 621 chicken proteins were detected as adsorbed to intestinal contents. Out of these, six chicken proteins were differently abundant in pellets obtained from *E. coli* or *E. faecium* associated chickens. Igλ, PIGR, pancreatic lipase PINLIP and CPA2 carboxypeptidase A2 were more abundant in the pellets obtained from caecal contents of *E. coli* associated chickens than in *E. faecium* colonised chickens, and CBS cystathionine β-synthase and ANPEP carboxypeptidase N were more frequently detected in the pellets obtained from the chickens associated with *E. faecium* (Figure 2).

## Discussion

In this study we addressed the influence of gut microbiota on both gene expression and protein abundance in the caecum of germ-free and colonised chickens. The major difference in gene expression was in the expression of immunoglobulins. Immunoglobulins were not expressed in germ-free chickens, similar to a previous report in mice [17] and consistent with the absence of B-lymphocytes in the caecum of germ-free chickens [18]. The expression of immunoglobulins was absolutely dependent on the presence of viable microbiota as heat killed microbiota did not trigger antibody production. This is also consistent with our previous observations on time dependent IgY and IgA expression in the chicken caecum. Newly hatched chickens did not express immunoglobulins in the caecum and expression of immunoglobulins in the caecum could be detected for the first time in the second week of life after gradual colonisation with microbiota [19, 20]. Interestingly, different microbiota had a different potential to stimulate antibody production. Tetraflora and complex conventional microbiota induced similarly high antibody production, higher than that induced by *E. coli* Nissle. However, *E. coli* Nissle stimulated antibody production to a higher extent than Gram-positive E*. faecium* DSM7134. This observation is similar to that of Pollard and Sharon who observed no development of Peyer’s patches in *Streptococcus* associated mice but recorded development of germinal zones in *Salmonella* Paratyphi A associated mice [21]. Likely, LPS from the Gram-negative *E. coli* (despite semirough structure of LPS in *E*. *coli* Nissle) or *Salmonella* acted as a potent stimulatory signal activating infiltration and development of B-lymphocytes although we must reiterate that the presence of LPS itself is not enough to trigger immunoglobulin expression since killed tetraflora failed to stimulate transcription of immunoglobulin genes. Contact and/or partial enterocyte invasion by live bacteria is therefore required for the induction of immunoglobulin expression. Higher expression of immunoglobulins in *E. coli* Nissle than in *E. faecium* DSM7134 colonised chickens was confirmed also in the pellets obtained from the caecal contents. Consistent with immunoglobulin translocation across the gut epithelium, we also detected a higher amount of polymeric immunoglobulin receptor protein (PIGR) bound to microbiota in *E. coli*-associated chickens than in *E. faecium* colonised chickens.

The system involved in sensing the presence of bacteria that was then translated into immunoglobulin production remains unclear. However, induction of IL1β, IL17 and IL22 could be part of this sensing and signalling pathway [9]. The induction of IL22 in response to gut microbiota may result also in an increased innate resistance since IL22 plays a profound role in the maintenance of epithelial integrity as well as in increased production of antimicrobial peptides [22]. Induction of PAPSS2 may be also indicative of increased innate resistance since 3-phospho-5-adenylyl sulfate produced by PAPSS2 is used as a donor of the sulfonate group for sulfatation of mucins forming a protective layer above the host epithelium [23].

Multiple additional processes were affected by gut colonisation although the differences in gene expression in germ-free and colonised chickens were quite moderate. Colonisation of the chicken caecum with gut microbiota affected the abundance of focal adhesion and extracellular matrix proteins. The decrease in all three subunits of collagen VI was recorded also in our previous study in which newly hatched chickens were colonised with microbiota from adult hens [12]. Moreover, downregulation of collagen VI was observed also in the chickens exposed to microbiota present in used litter [24] and collagen VI was shown to be the regulator of the focal adhesions in intestinal epithelial cells [25]. However, the difference in abundance of focal adhesion and extracellular matrix proteins in the current study was not observed at the level of transcription. Collagen VI is therefore transcribed and translated at the same rate in the colonised and germ free chickens and its differential abundance at protein level might be caused for example by different activity of collagen VI degrading proteases.

Gut colonisation likely resulted in a decrease in redox potential in the caecal tissue. This was indirectly documented by an increased abundance of enzymes required for fermentation such as GPI or LDHA. Colonisation may also led to an increased control of the production of reactive oxygen species by induction of CLIC5 which contributes to a decreased generation of reactive oxygen species in mitochondria [26]. An excess of reactive oxygen species could be controlled also by increased expression of TST which participates in neutralisation of sulphides produced by microbiota but also protects chicken tissue against reactive oxygen species [27]. Proposed gut anaerobisation resulted also in higher expression of TXNDC5 which is induced by hypoxia in endothelial cells [28] and is required for angiogenesis [29]. Gut colonisation resulted also in the induction of ASS1 which is part of citrulline recycling to arginine. NO radicals produced from recycled arginine by eNOS after gut colonisation then controls peristalsis or angiogenesis [30]. Anaerobisation of the caecal environment may therefore lead to physiological hypoxia associated with signals towards angiogenesis.

CBS, SULT1C3, SULT1E1 and PAPSS2 may belong to a group of functionally similar enzymes. CBS, cystathionine β-synthase converts homocysteine and serine to cystathionine which can be further converted to cysteine and taurine. Taurine is conjugated to bile acids in the liver [31]. Conjugated bile salts are released from the gall bladder to the duodenum but when reaching the distal ileum, most of the bile salts are deconjugated by microbiota [7, 8]. Bile salts are therefore resorbed in the deconjugated form and sulfotransferase, e.g. SULT1C3 or SULT1E1 may conjugate sulphate to deconjugated bile salts in epithelial cells following bile salt resorption. Sulfotransferases use 3-phospho-5-adenylyl sulfate as the donor of sulfonate, consistent with microbiota dependent induction of PAPSS2. Interestingly, CBS was more abundant both in caecal tissue and caecal contents of *E. faecium* DSM7134 than of *E. coli* Nissle colonised chickens. Different bacteria have a different potential for taurine deconjugation from bile acids [7], however, whether this is a correct explanation of this observation will have to be determined in future experiments.

Our results showed that colonisation of the chicken intestinal tract with gut microbiota triggers immunoglobulin expression and that the range of immunoglobulin expression was dependent on microbiota composition. Changes in additional processes like in the composition of extracellular matrix proteins, adaptation to reduced redox potential or sulfate conjugation were less extensive than immunoglobulin production but at least some of these processes were also dependent on microbiota composition. Gene and protein expression in the chicken caecum is therefore dependent on and influenced by microbiota composition.

## Additional files

**Additional file 1.** **List of primers used in quantitative RT PCR in the study.**

**Additional file 2.** **Differentially expressed proteins in at least one experimental group inoculated with live bacteria compared to expression in germ-free chickens.** There were 149 proteins differentially expressed in the caecum of germ-free and colonised chickens. Fifty the most induced or suppressed proteins are presented in Table 2 and all 149 differentially expressed proteins are listed.
